# Rapid and Sensitive Detection of Bacteria Response to Antibiotics Using Nanoporous Membrane and Graphene Quantum Dot (GQDs)-Based Electrochemical Biosensors

**DOI:** 10.3390/ma10060603

**Published:** 2017-05-31

**Authors:** Weiwei Ye, Jiubiao Guo, Xianfeng Bao, Tian Chen, Wenchuan Weng, Sheng Chen, Mo Yang

**Affiliations:** 1 Institute of Ocean Research, Zhejiang University of Technology, Hangzhou 310014, China; yeweiwei@zjut.edu.cn; 2 Department of Food Science and Technology, Zhejiang University of Technology, Hangzhou 310014, China; baoxianfeng950407@hotmail.com (X.B.); tsuinghatake0919@163.com (T.C.); 3 Interdisciplinary Division of Biomedical Engineering, The Hong Kong Polytechnic University, Hong Kong 00852, China; 4 Shenzhen Key Lab for Food Biological Safety Control, Food Safety and Technology Research Center, Hong Kong PolyU Shenzhen Research Institute, Shenzhen 518063, China; guojiubiao@163.com; 5 Department of Applied Biology and Chemical Technology, The Hong Kong Polytechnic University, Hong Kong 00852, China; 6 Guangdong Entry-Exit Inspection and Quarantine Bureau, Guangzhou 510623, China; wengvin@iqtc.cn

**Keywords:** nanoporous alumina membrane, graphene quantum dots, bacterial response to antibiotics, rapid, sensitive, electrochemical biosensor

## Abstract

The wide abuse of antibiotics has accelerated bacterial multiresistance, which means there is a need to develop tools for rapid detection and characterization of bacterial response to antibiotics in the management of infections. In the study, an electrochemical biosensor based on nanoporous alumina membrane and graphene quantum dots (GQDs) was developed for bacterial response to antibiotics detection. Anti-*Salmonella* antibody was conjugated with amino-modified GQDs by glutaraldehyde and immobilized on silanized nanoporous alumina membranes for *Salmonella* bacteria capture. The impedance signals across nanoporous membranes could monitor the capture of bacteria on nanoporous membranes as well as bacterial response to antibiotics. This nanoporous membrane and GQD-based electrochemical biosensor achieved rapid detection of bacterial response to antibiotics within 30 min, and the detection limit could reach the pM level. It was capable of investigating the response of bacteria exposed to antibiotics much more rapidly and conveniently than traditional tools. The capability of studying the dynamic effects of antibiotics on bacteria has potential applications in the field of monitoring disease therapy, detecting comprehensive food safety hazards and even life in hostile environment.

## 1. Introduction

The emergence of antibiotics therapy has led to a medical revolution in infectious disease treatment since the early twentieth century. The applications of antibiotics as therapeutic and preventive agents, together with vaccination and wide attention to hygienic practices, have resulted in a steady decrease in the morbidity and mortality associated with infectious diseases [1]. Such advances in infectious disease treatment boost the applications of antibiotics. The abuse of antibiotics for nonbacterial infections in recent years has resulted in multidrug-resistant bacteria pathogens, which are serious threats to the efficient treatment of diseases with these drugs [2]. Antibiotics are overused as the feed additives for growth promotion in livestock and poultry raising, plant production and aquaculture, having a detrimental effect on human health by direct contact on farms or via the food chain [3,4]. The consumption of meat or vegetables with antibiotic residues leads to antibiotic accumulation in human body, leading to toxicity to organs, hearing loss or diarrhea that is caused by disruption of the species composition in the intestinal flora [5]. Therefore, it is very necessary to develop techniques for quick and sensitive detection and characterization of bacterial response to antibiotics in the management of infections and food safety.

Chromatography-based techniques, such as gas chromatography with mass spectrometry (GC-MS), high-performance liquid chromatography (HPLC) and liquid chromatography with fluorescence (LC-fluorescence) have been developed for detecting antibiotics [6,7,8]. However, the complex procedures of chromatographic methods and requirement of highly trained technical persons have limited their wide applications. To circumvent these shortcomings, various detection methods have been successfully established, including diode array, flame ionization, and enzyme-linked immunosorbent assay (ELISA), for the detection of antibiotics residues [9,10,11]. These are suitable alternatives for antibiotics detection with accuracy and precision but not perfect candidates for characterizing bacterial response to antibiotics. Polymerase chain reaction (PCR)-based methods, myco-bacterial growth indicator tubes and resazurin-reduction assays have been developed for investigating bacterial response [12,13,14]. However, many of these various strategies are relatively high cost and time-consuming. Some traditional techniques require at least 24 h to complete the detection and characterization of bacterial response to antibiotics, so it is necessary to have fast and low-cost tools to investigate the drug susceptibility of bacteria for competing infections worldwide. Biosensors appear to be suitable or complementary analytical tools for investigating bacterial response to antibiotics due to their advantages of high selectivity, rapid detection, and in situ applications. Recently, there has been a rise in the fabrication of optical biosensors, surface-enhanced Raman scattering (SERS) sensors and electrochemical biosensors [15,16,17]. Impedance biosensors have become popular in the area of label-free and rapid bacteria and antibiotics detection [18,19]. Microfabricated interdigitated gold electrode impedance biosensors detected pathogenic bacteria via immobilized antimicrobial peptides [20]. An all-polymer electrochemical microfluidic biosensor using aptamers as recognition elements with a conductive polymer bilayer as an electrode material has been developed for sensitive antibiotics detection [21]. These impedance biosensors are time-saving and sensitive, but they are restricted and limited by the complexed fabrication procedures, high cost and electrode properties. Therefore, nanomaterials based biosensors are increasingly developed for the detection of small molecules or sensing of biological response in biomedical areas.

Nanomaterials are one of the attractive materials for the construction of biosensors in view of their excellent electronic, optical, and mechanical properties [22]. Nanoporous membranes with through nanopores have been used to construct electrochemical biosensors for proteins, bacteria cells and cancer cells detection with high sensitivity [23,24]. The sensing mechanism is based on monitoring impedance increase due to the ion current blockage through the nanopores because of molecules captured in the nanopores. With the strengths of high surface-to-volume ratio and easy fabrication with low cost, nanoporous membranes have wide applications in food toxin investigation, environmental monitoring and medical diagnosis [25,26,27]. Graphene quantum dots (GQDs) are graphene fragments smaller than 20 nm and show unique properties, such as remarkable conductivity, large surface-to-volume ratio, abundant hydrophilic edges as well as easy surface functionalization and low toxicity [28,29]. The increase in GQD geometric surface area enhances the interactive contact area with some electroactive analytes, so GQD modification on various substrates could improve the electrochemical reaction rate. These properties make GQDs excellent candidates for constructing nanoscaled electronic devices for various biosensing applications [30,31].

In the study, electrochemical sensing platforms based on nanoporous alumina membranes and GQDs have been developed for rapid and sensitive detection of bacterial response to antibiotics including enrofloxacin and ampicillin. They were used on the *Salmonella* bacteria that were captured on nanoporous membranes by reaction with antibody-GQD conjugation immobilized on membrane surfaces and nanopore walls. Bacterial responses to antibiotics were detected and characterized by the electrochemical biosensor system based on nanoporous alumina membranes and GQDs. The function of enrofloxacin and ampicillin on bacteria could kill live bacteria on the membranes, leading to impedance decrease. This nanoporous membrane combined with the GQD system allows the investigation of bacterial response to antibiotics with a simple, rapid and highly sensitive approach. The antibiotics could be rapidly detected within 30 min with the limit of detection (LOD) for enrofloxacin and ampicillin down to the pM level. This electrochemical biosensor with nanoporous alumina membrane and GQDs provides a new method for bacteria response to antibiotics investigation.

## 2. Results and Discussion

### 2.1. Mechanism of Bacterial Response to Antibiotics Detection

**Figure 1:**
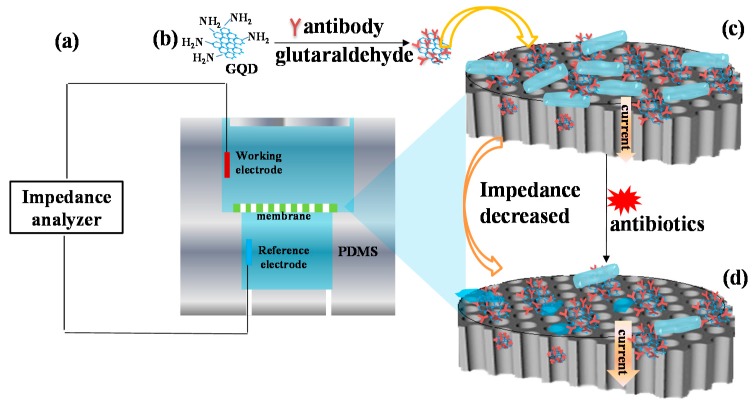
The schematic diagram of nanoporous membrane and GQD-based biosensor for *Salmonella* bacteria response to antibiotics detection.

The sensing mechanism and experimental processes are shown in Figure 1. The set-up consisted of a polydimethylsiloxane (PDMS) chamber integrated with a biofunctionalized nanoporous alumina membrane in the middle of the chamber as shown in Figure 1a. The nanoporous alumina membrane separated the PDMS chamber into an upper chamber and bottom chamber. Two platinum electrodes were inserted in the upper chamber and bottom chamber across the membrane as the working electrode and reference electrode, respectively. Impedance changes of bacteria capture on the nanoporous membrane and their response to antibiotics were recorded by the sensing system. Nanoporous alumina membranes were silanized by (3-glycidoxypropyl) trimethoxysilane (GPMS) for antibody immobilization. Amino modified GQDs were conjugated with antibody with glutaraldehyde as the linker (Figure 1b). They were immobilized on nanoporous alumina membranes by the reaction of epoxy groups on the membrane surface and amino groups of antibodies (Figure 1c). Bacteria were captured by antibodies on the membrane surface and in the nanopores to block ion flow through nanopores. The current was relatively low and the impedance was large as impedance had a ratio that was the inverse of the current. When antibiotics such as enrofloxacin and ampicillin were added and functioned on the *Salmonella* bacteria for minutes, the bacteria were deformed and became small in size. Therefore, the blocking effect for ion flow through nanopores decreased and current increased. When the impedance spectra were recorded, they decreased as the impedance ratio was the inverse of the current (Figure 1d).

### 2.2. Characterization of GQDs and Nanoporous Alumina Membranes

**Figure 2:**
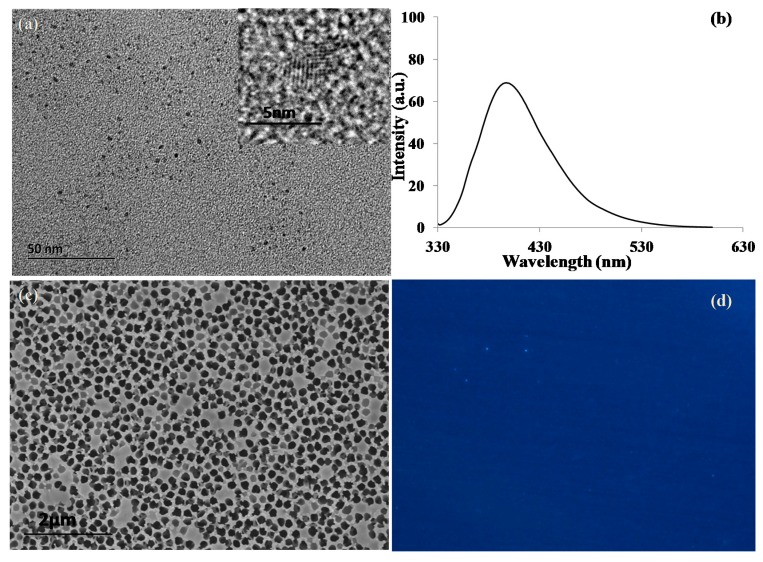
(**a**) TEM image of GQDs; (**b**) fluorescence spectroscopy of GQDs under 320 nm excitation in deionized (DI) water; (**c**) SEM image of antibody-GQD conjugation covalently linked on nanoporous alumina membrane; (**d**) fluorescence image of nanoporous alumina immobilized with antibody-GQDs conjugation under UV excitation.

The morphology and size of GQDs were observed by transmission electron microscopy (TEM) with the result shown in Figure 2a. GQDs with a small diameter of about 4 nm were dispersed homogeneously in the solution. The high-resolution TEM image of a single GQD (Figure 2a inset) showed the lattice fringe and the d-spacing of a single GQD was observed and estimated to be about 3.3 Å, which corresponded to the (002) plane of graphite [32]. The optical performance of GQDs exhibited a broad fluorescence emission wavelength ranging from 360 nm to 450 nm with the peak at 400 nm under excitation at 320 nm (Figure 2b). Figure 2c showed the scanning electron microscopy (SEM) image of antibody-GQD conjugation immobilized on the silanized nanoporous alumina membrane. Antibody-GQD conjugation was distributed on the surface of nanoporous alumina membrane for capturing bacteria cells. Fluorescence microscopy imaging was applied to characterize nanoporous alumina membrane immobilized with antibody-GQD conjugation. Under UV excitation, blue light emission was observed on the membrane, which further demonstrated successful immobilization of antibody-GQD conjugation on the nanoporous alumina membrane (Figure 2d).

**Figure 3:**
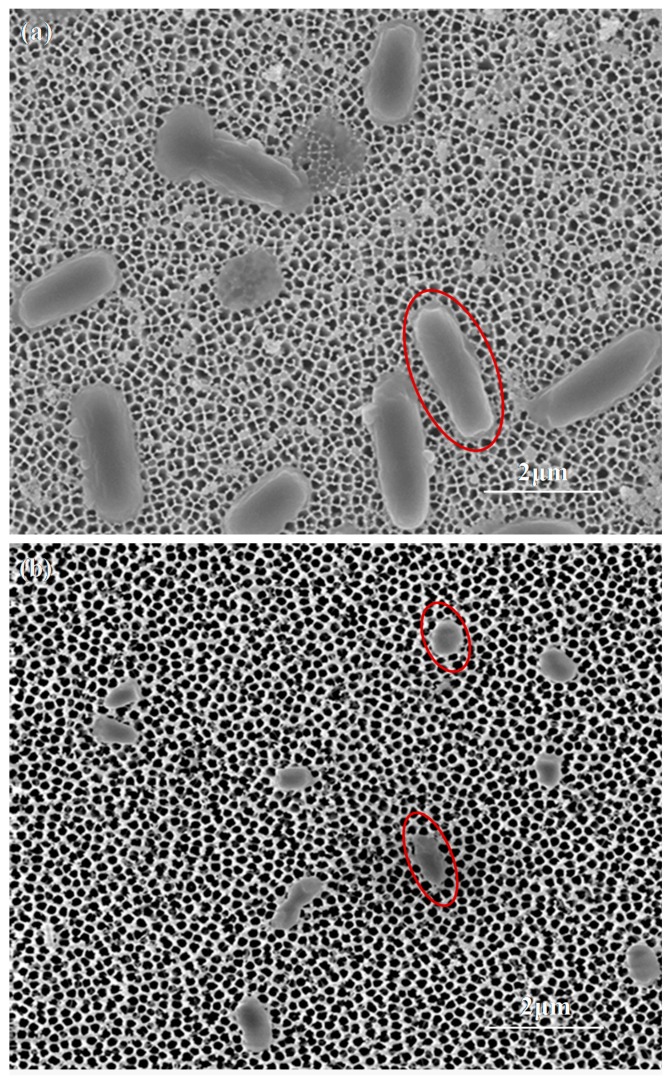
(**a**) SEM image of bacteria captured on nanoporous alumina membrane; (**b**) SEM image of bacteria after antibiotics treatment on nanoporous alumina membrane. The red ellipses indicated the shape change before and after antibiotics treatment.

To demonstrate *Salmonella* bacteria capture on the nanoporous alumina membrane and the antibiotic effect on bacteria, SEM images were obtained. When bacteria were captured on the nanoporous membrane, *Salmonellae* with 3D rod shapes, whose lengths were around 2 μm and diameters were around 1 μm, were clearly seen, as shown in Figure 3a. With antibiotic treatment of the bacteria, the bacteria shapes were deformed. The morphology changed from rod shapes to short or round shapes and they became smaller (Figure 3b). The bacteria underwent characteristic morphological changes with antibiotic treatment. It has been proposed that penicillin specifically leads to a fragile cell wall by affecting the link between the neighboring subunits [33]. The bacterium cell wall was mechanically weak. During antibiotic treatment, an increase of cytoplasmic mass acted on the mechanically weakened cell wall, increasing mechanical-osmotic pressure. The combined mechanical-osmotic pressure from inner eventually disrupted the cell wall, leading to death and lysis of the bacterium [34]. 

### 2.3. Impedance Sensing of Bacteria Capture on Nanoporous Alumina Membrane

**Figure 4:**
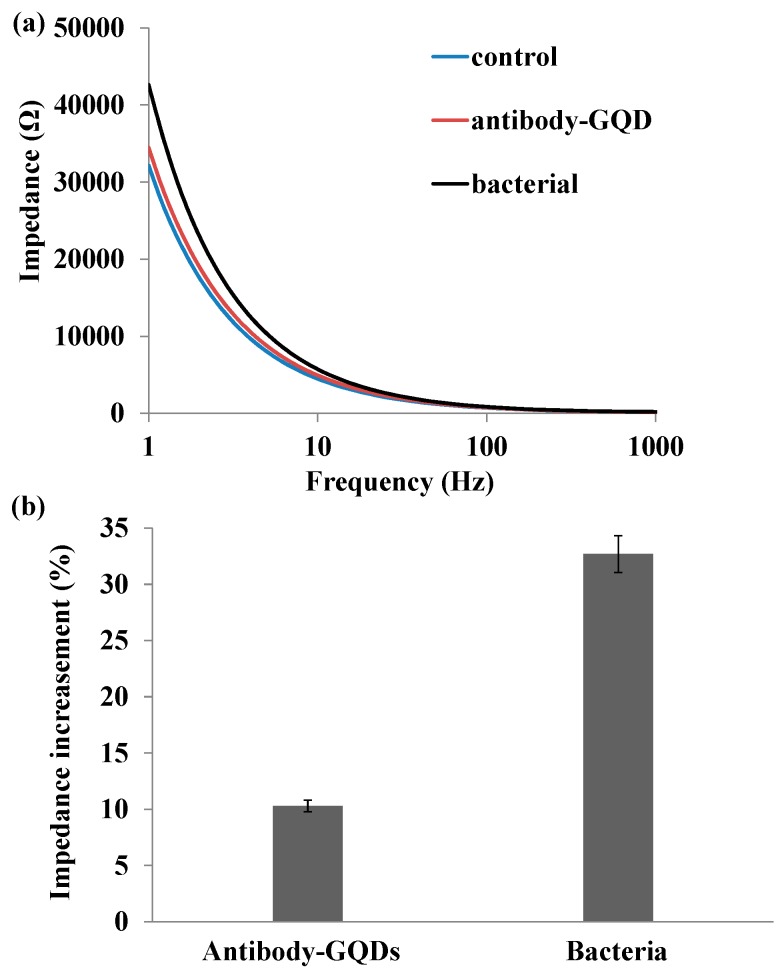
(**a**) Impedance spectra and (**b**) impedance amplitude change of nanoporous alumina membrane immobilized with antibody-GQDs conjugation and bacteria capture.

Impedance spectra were recorded to monitor *Salmonella* bacteria capture on a functionalized nanoporous alumina membrane. Figure 4a represented the impedance spectra of the nanoporous alumina membrane after surface modification, antibody-GQD conjugation covalently linked on nanoporous membrane and bacteria capture by the antibody in the frequency range from 1 Hz to 1 kHz. Impedance spectroscopy of nanoporous alumina membrane after surface modification was the control. PBS solution was injected in the upper chamber and bottom chamber at a rate of 5 μL/min by a syringe pump to fill in the chamber. The working electrode and reference electrode were immersed in the PBS solution to record impedance spectra across the nanoporous membrane. When antibody-GQD conjugation was immobilized on membrane and bacteria were captured, PBS solution was injected in the chamber to rinse off the non-specific binding of molecules on the membrane before impedance measurement. Impedance increased about 10% with antibody-GQDs conjugation immobilization on nanoporous alumina membrane, and 32% with bacteria capture compared with GPMS-modified nanoporous membrane (Figure 4b). The small amplitude increase about 10% with antibody-GQD conjugation showed the slight non-specific conjugation on membrane. The BSA blocking on membrane and GQDs decreased the non-specific adsorption of antibody on the membrane, obviously, which would have no effect on the bacteria detection signal. The bacterium is larger than the nanopores. The capture of bacteria on the membrane could significantly block the nanopores, leading to blocking of ion flow through the nanopores. Therefore, impedance amplitude presented a significant increase with bacteria capture on the nanoporous alumina membrane.

### 2.4. Impedance Sensing of Bacteria Cells Response to Antibiotics

**Figure 5:**
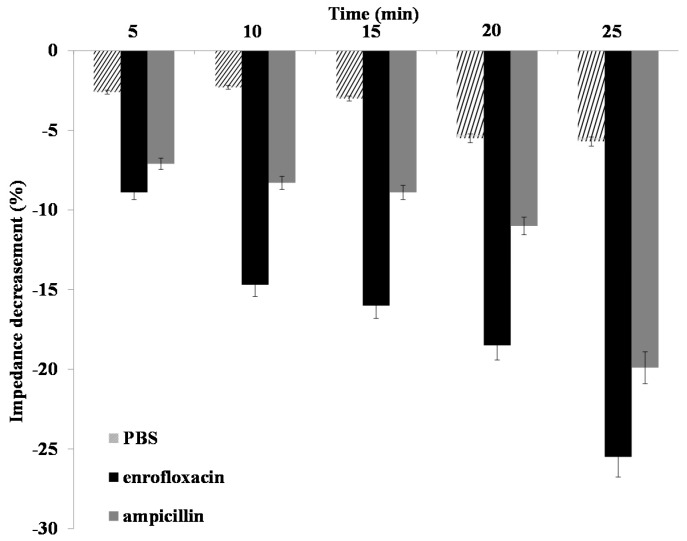
Time courses of the relative impedance amplitude signal changes of the nanoporous alumina membrane with GQDs with antibiotics function time.

Nanoporous alumina membranes with *Salmonella typhimurium* bacteria (10^4^ cfu/μL) were used for bacteria response to antibiotics detection. To demonstrate the functionality of this biosensor for antibiotics response investigation, two kinds of antibiotics solution, including enrofloxacin and ampicillin, with a concentration of 0.1 nM were added to operate on the *Salmonella* bacteria at room temperature. Impedance spectra were recorded after injection of PBS solution into the PDMS chamber to wash away the antibiotics residues to eliminate interference with the electrolyte. Figure 5 showed the impedance amplitude changes across the nanoporous membrane with function time slots of 5 min, 10 min, 15 min, 20 min and 25 min after addition of enrofloxacin and ampicillin. Enrofloxacin, a second generation of fluoroquinolones, is used as a veterinary medicine for the treatment of salmonellosis because of its strong antibacterial properties and effective diffusion across cells. Ampicillin, the first broad-spectrum penicillin, is widely used to prevent and treat a great number of bacterial infections. With the function of enrofloxacin and ampicillin on bacteria, the impedance amplitude decreased as the time increased. The addition of PBS to bacteria was used as the control group, with the results also shown in Figure 5. It had no obvious change with the increase in incubation time. The impedance amplitude decreased the effectiveness of enrofloxacin much more than that of ampicillin at 5 min, 10 min, 15 min, 20 min and 25 min, showing a more sensitive response from *Samonella* bacteria to enrofloxacin.

**Figure 6:**
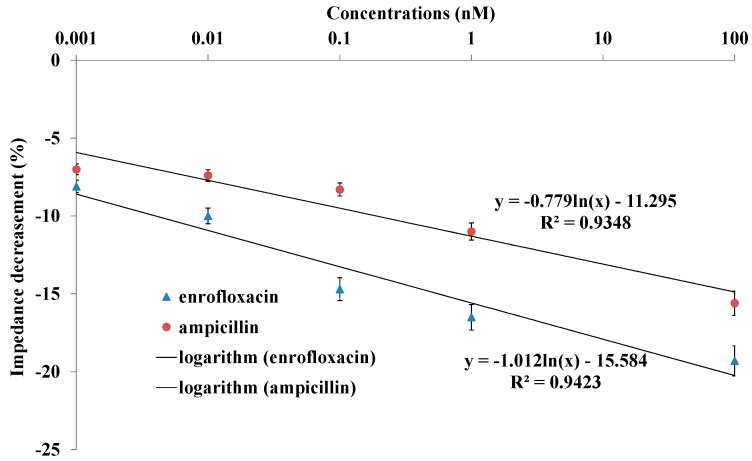
The impedance change of nanoporous alumina membrane with GQDs with different concentrations of enrofloxacin and ampicillin at incubation time of 10 min.

To explore the detection of bacterial response to various antibiotics concentrations, further experiments with various antibiotics concentrations were conducted on the sensing platform. Figure 6 showed the impedance decreases with antibiotics concentrations ranging from 1 pM to 100 nM. All of the different concentrations caused the relative impedance change, showing an obvious antibiotic effect that was dependent on concentrations. Impedance decreased with the increase of enrofloxacin and ampicillin concentrations. For the bacterial response to antibiotics, a linear relationship was found between the relative impedance signal decrease and logarithm concentrations of enrofloxacin and ampicillin with the corresponding equations *y* = −1.012ln(*x*) − 15.584 with *R*^2^ = 0.9423 and *y* = −0.779ln(*x*) − 11.295 with *R*^2^ = 0.9348, respectively. The LOD was around 1 pM and 40 pM for enrofloxacin and ampicillin detection, respectively. It was calculated from the equations based on control signals plus three times the noise signal (standard derivation). 

In order to emphasize the advantages of nanoporous alumina membranes and GQD-based electrochemical biosensors, nanoporous alumina membrane-based electrochemical biosensors without GQDs were also investigated for detection of bacterial response to antibiotics. Antibodies (0.5 μg/mL) were immobilized on GPMS-modified nanoporous alumina membrane and bacteria were captured. When enrofloxacin and ampicillin affected the bacteria, the bacteria were deformed. They were observed and evaluated by impedance spectra. With the addition of PBS to bacteria as the control, impedance amplitude changes with addition of 10 nM enrofloxacin and ampicillin at 5 min, 10 min, 15 min, 20 min and 25 min were found, as shown in Figure S1. The maximum decrease of impedance amplitude was about 10% with antibiotics function for 25 min. Figure S2 showed the impedance decrease with concentrations of enrofloxacin and ampicillin ranging from 1 nM to 100 µM. Impedance decreased from 1.7% to 10% and 1.5% to 8.3% for enrofloxacin and ampicillin, respectively. The relatively large antibiotics concentrations did not cause an obvious impedance decrease. Therefore, nanoporous alumina membrane and GQDs-based electrochemical biosensor presented improved sensitivity and low LOD in comparison with nanoporous alumina membrane based electrochemical biosensor for bacteria response to antibiotics detection without GQDs. 

In the study, GQDs with single-atom-thick planar sheets and a *sp*^2^-hybridized carbon structure perfectly arranged in a honeycomb lattice were used to increase the surface area, and provide interfaces with sites for much more antibodies conjugation and *Salmonella* bacteria capture on nanoporous alumina membranes, laying the foundation of outstanding analytical performances. GQDs may also act as efficient antibiotics carriers due to their π-π stacking interaction, high surface-to-volume ratio area and drug loading capacity. Therefore, antibiotics could continuously release from GQDs to kill the bacteria. This developed nanoporous alumina membrane and GQDs based electrochemical biosensor was suitable for rapid, sensitive and convenient detection of bacterial response to antibiotics by impedance spectroscopy. It enables monitoring of the bacteria response to antibiotics in real time. It provides a sensing platform for studying the dynamic effects of multiple antibiotics on bacteria and even detecting life in hostile environment. The sensing platform can be used to establish biosensor models for potential applications in the field of monitoring disease therapy and comprehensive toxicity investigation in rapid food safety detection.

## 3. Materials and Methods 

### 3.1. Nanoporous Alumina Membrane Surface Functionalization

Nanoporous alumina membranes with 200 nm nanopore diameter and 60 µm thickness (Whatman, Inc., Maidstone, UK) were functionalized to detect bacterial response to antibiotics. Nanoporous membranes were modified by GPMS (98%, Sigma-Aldrich, Darmstadt, Germany) for antibody conjugation according to the procedures described in the previous study [26]. In brief, membranes were boiled in hydrogen peroxide (H_2_O_2_, 30%, Sigma-Aldrich) for 30 min. They were washed in DI water for 10 min and dried. Then, they were silanized by GPMS in the mixture of toluene and GPMS (2%) at 60 °C overnight. The membranes were rinsed by toluene (99.8%, Sigma-Aldrich) and followed by anhydrous ethanol (99.8%). It repeated for three times. The membranes were cured for 2 h at 60 °C and the functional epoxy groups formed on the membrane surfaces.

### 3.2. PDMS Chamber and Functionalized Nanoporous Membrane Integration

A PDMS chamber was made from a 184 silicone elastomer kit (Dow Corning Corp., Midland, MI, USA) with the mixture of silicone elastomer base and the curing agent (10:1). The mixture was stirred thoroughly and transferred into a mould and degassed. It was put into an oven at 95 °C for thermal curing. In 1 h, the chamber was separated from the mould and cylindrical cavity of different diameters formed in the chamber. The PDMS chamber was treated by oxygen plasma to clean the chamber and improve the bonding of nanoporous alumina membrane and the chamber. The functionalized nanoporous alumina membrane was then anchored between cylinders in the chamber and bonded by infiltration of the silicone elastomer base and curing agent mixture with curing at 60 °C for about 1 h in an oven. 

### 3.3. Anti Salmonella Monoclonal Antibody Preparation and Analysis

The monoclonal antibody of Salmonella was designed and developed using *Salmonella* membrane protein C (OmpC) as antigen. The monoclonal antibody was produced by following standard preparation procedures including immunization of mouse, hyperdoma screening and antibody screening [35]. The sera were collected from mouse immunized with OmpC and precipitated. The monoclonal antibody was purified by passing through protein A sepharose column. It was eluted with glycine-HCl buffer (0.1 M, pH 2.5) and neutralized with Tris (1 M, pH 8.0). The antibody was dialyzed in PBS (pH 7.4) and stored in aliquots at −20 °C for later use. The antibody was tested by using various serotypes of *Salmonella* and other bacteria types, such as *E. coli*, *Shigella* spp., *Klebsiella* spp., *Enterobacter* spp., *Pseudomonas* spp., *Citrobacter* spp., etc. The prepared antibody could specifically target to Salmonella. Moreover, the antibody had a broad *Salmonella* detection spectrum in recognizing about 32 *Salmonella* serotypes including *Salmonella Enteritis, Typhimurim*, etc. while did not react to other species of bacteria tested. 

### 3.4. Preparation of Biofunctional GQDs on Nanoporous Membrane

Amino modified GQDs with the average sheet size of about 4 nm were biofunctionalized by *Salmonella* monoclonal antibody with glutaraldehyde as the linker. GQDs (5 µL, 1 mg/mL) were mixed with glutaraldehyde (0.1 µL, 25%). The pH of the mixture was adjusted to pH 7.6 and vortexed. In half an hour, antibody (0.5 g/L, 10 µL) was added to the GQDs solution and incubated at 4 °C for about 4 h. The antibody-GQDs conjugation was diluted by PBS to the volume of 500 µL and added to 5 membranes with each membrane of 100 µL. It incubated for 4 h to make antibody-GQDs conjugation anchor on the nanoporous alumina membrane. Bovine serum albumin (BSA, 1%) in PBS (pH 8.0) was stayed on the membranes for another 4 h to reduce the non-specific adsorption.

### 3.5. Bacterial Cell Culture and Plate Counting

*Salmonella typhimurium* samples were separated from meat and grown in nutrient broth for 24–36 h at 37 °C. The concentrations of bacteria cells were calculated by the plate counting method. The cultured *Salmonella* bacteria cells were collected and diluted with PBS for several times. The diluted sample (1 mL) was placed in nutrient broth for incubation at 37 °C. In 24 h, each bacterium sample was divided to visible single colony. The concentration of original bacteria cells can be obtained by counting the colonies on the plate, and calculated by the following formula:

C = N/V × n,(1)

where C is the original concentration of bacteria cells (CFU/μL), N is the colony number obtained on the plate, V is the total volume of the initial bacteria cells (μL), and n is the times for dilution.

### 3.6. Electrochemical Impedance Spectroscopy Measurement

The cultured *Salmonella typhimurium* bacteria were captured by antibody-GQDs conjugation that was immobilized on the nanoporous alumina membranes, which were integrated in the PDMS chamber. Electrochemical impedance spectroscopy was measured via a two-electrode system with two platinum wire electrodes placed across the nanoporous alumina membrane in the PDMS chamber to record the spectra change of bacteria capture and their response to antibiotics with different concentrations and different function time. The impedance spectra were recorded by an electrochemical analyzer Autolab Potentiostat Galvanostat (Metroholm, Zofingen, Switzerland). The measurement process was controlled by NOVA 1.10 software package (Metroholm, Zofingen, Switzerland). The impedance signal changes were recorded and analyzed. 

### 3.7. Characterization

The size and morphology of GQDs were characterized by a TEM (JEOL-2100F, JEOL, Tokyo, Japan). Ultrathin layer of carbon coated copper grids were prepared. Samples (10 μL) were dropped on the copper grids and placed under light for quick drying for TEM characterization. Fluorescence images of nanoporous alumina membranes with antibody-GQDs conjugation were obtained by a fluorescence microscope (Nikon Eclipse 80i, Nikon, Tokyo, Japan). Fluorescence spectra of GQDs were recorded from F-2700 fluorescence spectrophotometer (Hitachi Ltd., Tokyo, Japan). The morphologies of bare nanoporous alumina membranes, bacteria on membranes and the morphology changes of bacteria with antibiotics treatment were characterized by SEM (JEOL model JSM-6490 SEM system, JEOL, Tokyo, Japan). The bacteria were captured on nanoporous alumina membrane by antibodies and treated with glutaraldehyde solution (3%) for 3 h at 10 °C. It was then dehydrated in 25%, 50%, 75% and 99% ethanol each for 10 min. The bacteria with bacteria treatment were also fixed by the above steps. The fixed bacteria were observed with SEM.

## 4. Conclusions

In summary, a nanoporous membrane combined with a GQD sensing platform was developed for rapid, sensitive and convenient detection of bacterial response to antibiotics by impedance spectroscopy. The nanoporous structure of the membrane and GQD significantly increases the surface-to-volume ratio and enhances biosensor sensitivity. The antibiotics detection can be finished in half an hour and the LOD for antibiotics can reach the pM level. The sensing platform is the foundation of establishing bacteria biosensor models for potential applications in the field of early disease diagnosis, detecting life in hostile environments and comprehensive toxicity investigation in rapid food safety detection. 

## Supplementary Materials

The following are available online at http://www.mdpi.com/1996-1944/10/6/603/s1, Figure S1: Time courses of the relative impedance amplitude signal changes of nanoporous alumina membrane without GQDs with the antibiotics function time; Figure S2: The impedance change of nanoporous alumina membrane without GQDs with different concentrations of enrofloxacin and ampicillin at incubation time of 10 min.
