# Supplementary material for: Rapid and Sensitive Detection of Bacteria Response to Antibiotics Using Nanoporous Membrane and Graphene Quantum Dot (GQDs)-Based Electrochemical Biosensors
Source: Materials (Basel). 2017 May 31;10(6):603. doi: 10.3390/ma10060603 (PMC5553420; doi:10.3390/ma10060603)
Supplement: Supplementary file 1 [file materials-10-00603-s001.pdf]

Supporting Information

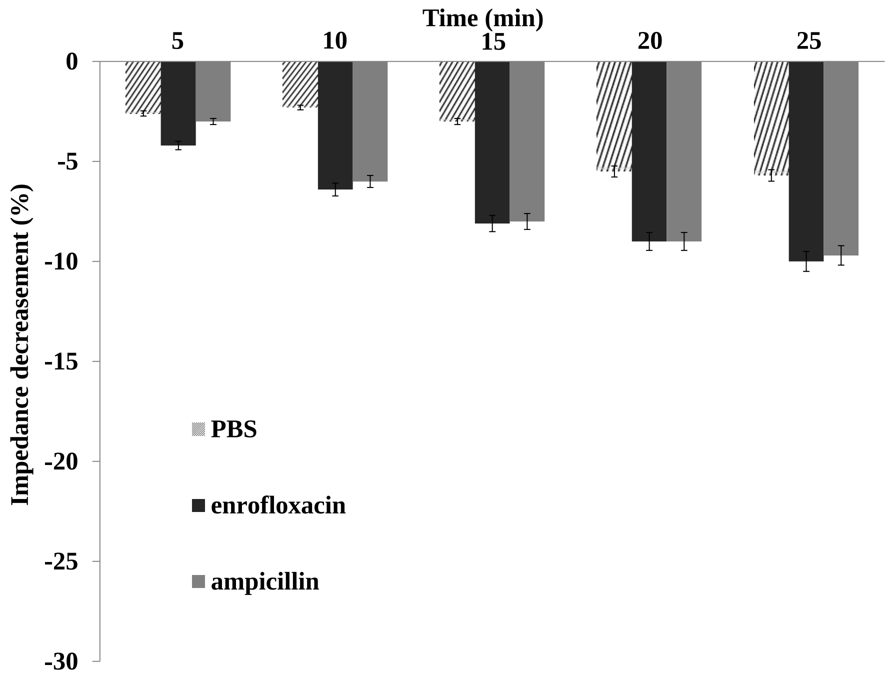

Figure S1. Time courses of the relative impedance amplitude signal changes of nanoporous alumina membrane without GQDs with the antibiotics function time.

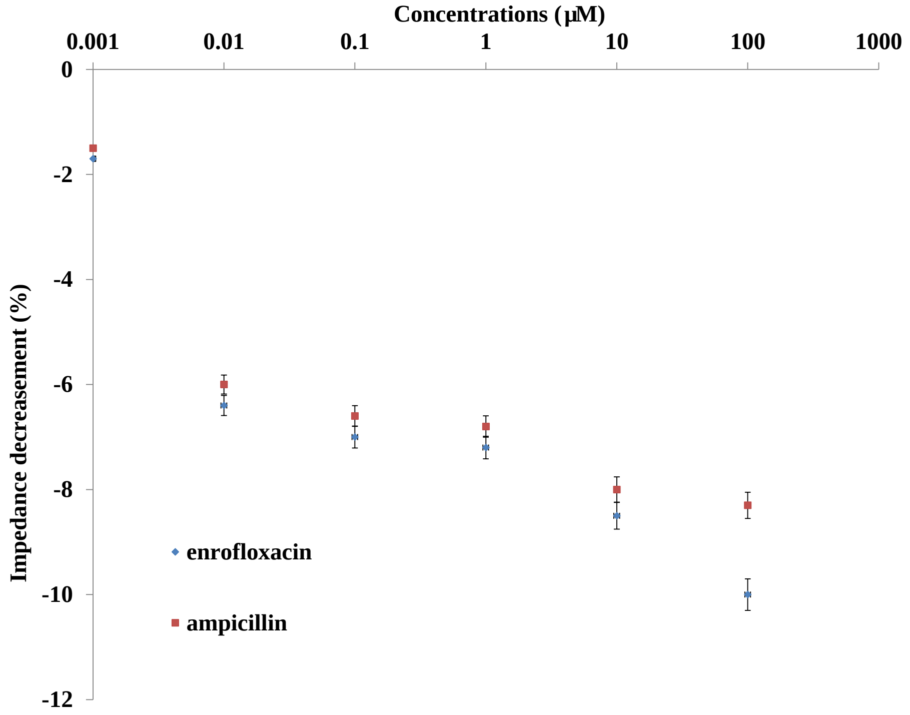

Figure S2. The impedance change of nanoporous alumina membrane without GQDs with different concentrations of enrofloxacin and ampicillin at incubation time of 10 minutes.
